# Utility-Based Multicriteria Model for Screening Patients under the COVID-19 Pandemic

**DOI:** 10.1155/2020/9391251

**Published:** 2020-09-01

**Authors:** Lucia Reis Peixoto Roselli, Eduarda Asfora Frej, Rodrigo José Pires Ferreira, Alexandre Ramalho Alberti, Adiel Teixeira de Almeida

**Affiliations:** Universidade Federal de Pernambuco, Av. Acadêmico Hélio Ramos, s/n-Cidade Universitária, Recife, PE CEP 50.740-530, Brazil

## Abstract

In this paper, a utility-based multicriteria model is proposed to support the physicians to deal with an important medical decision—the screening decision problem—given the squeeze put on resources due to the COVID-19 pandemic. Since the COVID-19 emerged, the number of patients with an acute respiratory failure has increased in the health units. This chaotic situation has led to a deficiency in health resources. Thus, this study, using the concepts of the multiattribute utility theory (MAUT), puts forward a mathematical model to aid physicians in the screening decision problem. The model is used to generate which of the three alternatives is the best one for where patients with suspected COVID-19 should be treated, namely, an intensive care unit (ICU), a hospital ward, or at home in isolation. Also, a decision information system, called SIDTriagem, is constructed and illustrated to operate the mathematical model proposed.

## 1. Introduction

At the start of the current year (2020), the COVID-19 disease, caused by the new coronavirus (the SARS-CoV-2), was deemed to be an epidemic. However, a few weeks later, it was reclassified as a pandemic. Since the COVID-19 has emerged, it has been present in different degrees of illness in the human organism, causing, in severe cases, acute respiratory failure [1–3].

In this context, this disease has resulted in an increasing number of patients requiring hospital treatments, especially in intensive care units (ICUs) with the support of mechanical ventilation equipment. Consequently, in many cities around the world, this increase in the demand for places in hospitals has placed a great strain on medical resources and revealed deficiencies in all forms of provision for a pandemic and therefore adequate treatment has not been available for all the severe cases.

Thus, to deal with this chaotic situation, it is fundamental to have decision-making strategies in place as these are important to ensure that the expectation of the survival of COVID-19 patients can be maximized. In other words, in such situations, which involves a risk context, it is important to conduct a rational decision-making process in order to be able to save the majority of patients [4].

Therefore, in order to allow the rational conduct of an important medical decision-making problem—the screening decision problem—a utility-based multicriteria model is proposed in this study.

This additive multicriteria decision-making model is based on the multiattribute utility theory (MAUT) approach [5], which considers the concepts of the utility theory [6], to deal with the uncertainty presented in medical diagnostics [7]. Thus, in this study, issues from operational research are considered to support a healthcare decision-making problem.

Also, a decision information system (DIS), called SIDTriagem, is constructed to implement this multicriteria decision-making model. The outcome of this DIS is a recommendation about where a patient with suspected COVID-19 should be directed to, considering the alternatives presented in the screening problem. It is worth mentioning that this model is a supplement to support physicians to deal with the screening problem. It is left to the physicians to decide whether or not to follow the recommendation made.

This paper as organized as follows.  Section 2 details the screening decision problem.  Section 3 describes the utility-based model proposed in this study.  Section 4 presents the applicability of the proposed approach and discusses these results. Finally, Section 5 draws some conclusions and indicates possible topics for future lines of research.

## 2. The Screening Decision Problem

The COVID-19 pandemic has provoked several decision situations that physicians are facing in their routines in hospitals and other health units all over the world. One of the main decision problems that they encounter every day is how best to screen patients with suspected COVID-19. A patient arrives at a health unit with symptoms and other complaints. Then, the physician has to decide, depending on the patient's clinical state, whether this patient should be sent for treatment in an intensive care unit (ICU), in a hospital ward (but not in an ICU) or whether he/she should be sent home to isolate instead of being hospitalized. This is the classical screening problem that is addressed in this paper, in which the context of the COVID-19 pandemic has been made more acute. Given that uncertainty is an inevitable factor that is inherently present in medical diagnostics and treatment decisions [6], a decision analysis (DA) model based on the multiattribute utility theory (MAUT) is developed in order to aid physicians when they make such decisions.

DA support for screening patients within the context of other diseases has been widely explored in the literature. Xu et al. [8] used a DA approach based on decision trees to investigate strategies for the triage of patients with symptoms of acute stroke. Outcomes were measured based on workflow times. Probabilities and input parameters were estimated based on guidelines and previously published studies. A practical analysis was conducted using TreeAge Pro software. Jiang et al. [9] designed a DA tree in order to evaluate the best strategy for treating patients after an esophagectomy. The TreeAge Pro software was used to construct the decision tree. Two strategies were compared based on several factors, such as length of stay in the hospital, costs, and possible complications. A sensitivity analysis was performed by using a Monte-Carlo simulation. Felder and Mayrhofer [10] analyze the impact of risk preferences in decisions about medical screening, testing, and treatment. They conclude that a risk averse decision-maker tests and treats patients at lower probabilities of illness, compared to risk neutral and risk vulnerable decision-makers. Cleary et al. (2005) [11] applied DA techniques for comparing three different strategies of screening for herpes, a simple virus, in pregnant women; probability estimations were derived from DA on the literature. Kiberd and Forward [12] developed a DA-based study to investigate the impact of medical screening decisions for West Nile virus in organ transplantation, by considering lives lost and saved.

A cost-effective analysis approach was also widely used by authors when dealing with DA models for screening patients, and these studies covered a wide range of diseases. Wilson and Howe [13] developed a DA model for screening methods of dysphagia after stroke. Different strategies were compared based on a cost-effective analysis. Medical costs were measured from a societal perspective, and effectiveness was measured in years of quality-adjusted life. Sensitivity analysis using a Monte-Carlo simulation was performed. Donnan et al. [14] conducted a cost-effectiveness analysis for DA of children with acute lymphoblastic leukemia. Probabilities were obtained based on published evidence in the literature, and survival was measured in months of life. Cooper et al. [15] constructed a decision analysis model to handle health outcome states and costs of screening strategies for children in preoperative coagulation tests prior to a tonsillectomy and/or adenoidectomy. Probabilities, costs, and utility data were estimated based on a review of databases. Sensitivity analysis was performed so that parameters were widely varied. Baeten et al. [16] conducted a study to show the potential and impact of three approaches in the use of cost-effective analysis in the scope of breast cancer control: targeting specific groups, by comparing disparities; equity weighting, by valuing high and low health gains differently; and multicriteria decision analysis, giving weights for multiple equity and efficient criteria. Oh et al. [17] used DA based on a cost-effective approach to compare different strategies for screening rheumatoid arthritis and systemic lupus erythematosus patients. Data were obtained from previous studies and from real practical cases. Rulyak et al. [18] applied DA for screening strategies in familial pancreatic cancer kindreds. Life expectancy and lifetime medical care costs were modeled in order to conduct a cost-effective analysis. McGrath et al. (2002) [19] used DA software (TreeAgre Pro) for comparing four strategies for screening patients with colorectal cancer, taking into account the cost to find an advanced adenoma. Probabilities, test characteristics, and costs were estimated based on a literature review and local costs.

In this context, this paper is aimed at presenting a multicriteria model for screening patients with suspected COVID-19, based on a DA approach within the multiattribute utility theory. Two main factors are taken into account: the life of the patient being screened and the cost of the alternative indicated for that patient. These criteria are further detailed in this paper. Subjective probabilities are considered for the construction of a decision tree for the screening problem. The next section details the whole structure of the mathematical model proposed for the screening problem.

## 3. Utility-Based Model for Aiding the Screening of Suspected COVID-19 Patients

### 3.1. Decision Tree for the Screening Problem

In this section, the decision tree technique is used to illustrate the screening decision problem investigated in this study [9, 10]. According to Cheng et al. [20], this technique can be used to identify the risk factors presented in a decision-making problem, it being possible to consider the outcomes obtained by their combination.

In this context, in the decision tree constructed, the alternatives indicated to conduct the health treatment for the patients with suspected COVID-19 are identified. In the screening problem investigated in this study, three alternatives are considered; these alternatives are ICU stay (ICU), hospital stay (HS), and isolation at home (IH).

Also, for each one of these alternatives, the uncertainty is presented, since according to [6], uncertainty is an inevitable factor that is inherently present in treatment decisions. Thus, in an uncertainty context, the consequences to be obtained depend on the alternatives and the state of nature [6, 21].

In other words, for each alternative and each state of nature, which is represented by the chances to survive and chances to death, a consequence is obtained. For the alternative ICU stay, the patient can survive the ICU stay or die during the ICU stay. For the alternative hospital stay, the patient can survive the hospital stay or die during the hospital stay. Finally, for the alternative isolation at home, the patient can survive isolation at home or die during isolation at home.

**Figure 1:**
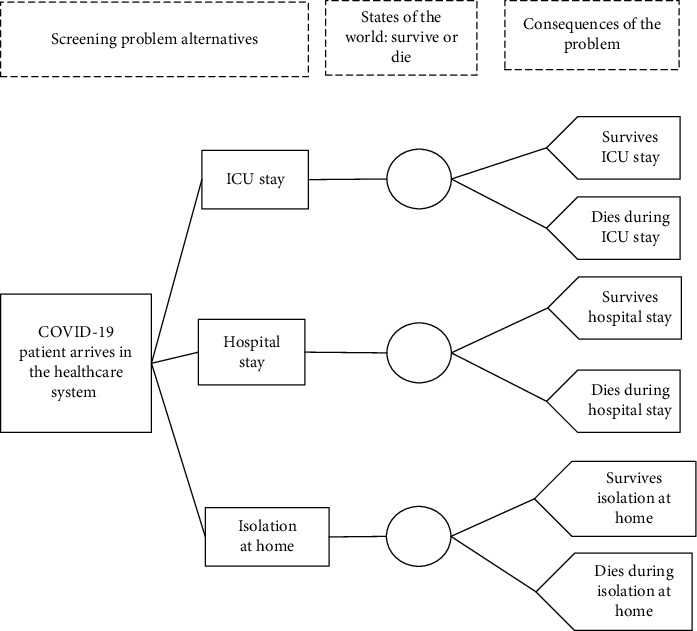
Screening problem decision tree.

It is worth mentioning that in the decision tree technique the squares are the decision nodes, the circles are the chance nodes, and the arrows are used to connect these decision elements [8]. In this context, in the decision tree constructed, four squares and three circles are presented. The decision tree constructed is illustrated in Figure 1.

Based on the decision tree illustrated in Figure 1, the mathematical model used to construct the utility-based multicriteria model is described in the next section. This mathematical model connected the decision elements presented in Figure 1 to obtain the recommendations (outputs) for the screening problem investigated.

### 3.2. Mathematical Model

In this section, the mathematical model, presented in the utility-based multicriteria model, is described. This mathematical model is based on the multiattribute utility theory (MAUT) [4] and takes into account the concepts of the utility theory [6] and multicriteria approach [5, 22, 23].

The utility theory [6] is a very appropriate way to deal with decision-making under uncertainty. In this context, states of the world (or states of nature) are used to represent the uncertainty presented in the decision scenario. Also, for each state of nature, probabilities are assigned to represent their chance of occurring, and these are obtained by an expert or by the decision-maker (from the subjective expected utility model) [21].

In this context, regarding the screening decision-making process considered in this study, the states of nature are survival or death, and the alternatives are the options to conduct the healthcare treatment with the patients with suspected COVID-19, with three alternatives being considered: ICU stay (ICU), hospital stay (HS) and isolation at home (IH).

Also, two criteria are considered in this complex decision situation, the life of the patient being screened and the cost of the alternative indicated for that patient. The cost of an alternative is subjectively related to the impact on the health system, considering resource constraints. Therefore, alternative “home isolation,” for example, presents no cost for the health system, since the patient will stay at home and no health resources will be occupied by this patient. For alternative “ICU stay,” however, the cost for the health system might be high, especially when ICU occupation is high and resources are scarce. The alternatives are evaluated in each one of these criteria, considering the multicriteria decision scenario [5, 22, 23].

Thus, for this decision-making problem, the decision-maker's preferences are assumed to be represented by MAUT [5]. In this context, from the corroboration of the additive independence condition, the additive aggregation analytic form is used to construct the mathematical model. The multiattribute utility function is presented in equation (1), where *a* is the alternative, *k*_*j*_ is the scaling constant for criterion *j*, and *u*_*j*_(*a*) is the marginal utility function in criterion *j*:

(1) $$\begin{array}{c} u\left(a\right)=\overset{n}{\underset{j=1}{\sum }}k_{j}u_{j}\left(a\right). \end{array}$$

It is worth mentioning that the scaling constants are obtained by applying an elicitation procedure with a decision-maker. The values of the scaling constants, for both criteria, are equal to 0.5 and their sum is equal to 1, in accordance with MAUT concepts [5]. The values of the scaling constants are presented in equation (2):

(2) $$\begin{array}{c} k_{\text{L}}=k_{\text{C}}=0.5. \end{array}$$

Also, the utility functions represent the consequences in each state of nature. For this study, the marginal utility functions are also obtained in the elicitation procedure. In this context, for the criterion patient's life, the utility functions are equal for the three alternatives, namely, 1 if the state of nature is survival and 0 if the state of nature is death. Equations (3) and (4) illustrate this condition:

(3) $$\begin{array}{c} u\left(\text{Shi}\right)=u\left(\text{Shs}\right)=u\left(\text{Sicu}\right)=1, \end{array}$$

(4) $$\begin{array}{c} u\left(\text{Dhi}\right)=u\left(\text{Dhs}\right)=u\left(\text{Dicu}\right)=0. \end{array}$$

On the other hand, regarding the criterion cost, the utility function is the same, since it does not depend on the state of nature. In this situation, the resources were consumed in the hope of saving the patient, regardless of whether the patient survives or dies.

In addition, for the criterion cost, the utility function for the alternative ICU stay presented the worst value, since the health treatment in ICU is more expensive. Analogously, for the alternative isolation at home, the utility function presented the highest value, it being the most desirable alternative [24–26].

An important consideration for the ICU stay utility function is the dependence regarding another variable, which is associated to the probability of a “future patient” arriving in the healthcare system, in a severe condition, and requiring to be sent to the ICU, combined with the occupation rate of the ICU. This variable is called Fp, this being an acronym of the probability of a future patient arriving in the healthcare system. If Fp is equal to 1, this indicates that no beds are available in the ICU. In this context, the utility function for this situation presents the worst value, this being a chaotic situation. In this scenario, it is difficult to accommodate patients in the ICU, with a tendency to recommend the alternatives hospital stay or isolation at home. As to these considerations, the utility function for an ICU stay, for the evaluation in the criterion cost, is defined according to equation (5):

(5) $$\begin{array}{c} u\left(\text{Cicu}\right)=1-\text{Fp}. \end{array}$$

For the worst case, when the ICU is completely occupied, Fp is equal to 1 (which means that sending the patient to the ICU would lead to a very high cost, thus leading to an utility equal to 0). In the opposite extreme case, Fp would be equal to 0. However, a parameterization for three possible cases between these two extreme situations is considered for the decision information system: high occupation (Fp = 0.7), intermediate occupation (Fp = 0.5), and low occupation (Fp = 0.3). In order to define these values of 0.7, 0.5, and 0.3, three doctors were consulted and simulations were performed in order to verify which values fit best according to doctors' actual attitude. It is worth mentioning, however, that these values depend on the decision-maker judgments, and in the SIDTriagem, these ranges can be adjusted if the user so desires.

The utility function for isolation at home, in the criterion cost, i.e., (*U*(Chi)), is equal to 1. Also, the utility function for hospital stay (*U*(Chs)) is between 0 and 1. In this study, the utility function equal to 0.8 is considered. However, a variation can be applied using the Monte-Carlo simulation, as presented in the next section.

Finally, another important variable to be considered in this mathematical model is the subjective probability assigned for each state of nature in order represent its chance of occurring. Thus, these probabilities are given by the physicians, considering their subjective evaluation about the patient's state of health.

In other words, the physician has to define a probability of surviving (chance of surviving) for the patient considering each one of the alternatives. These probabilities are represented by *π*(Shi), *π*(Shs), and *π*(Sicu); their sum being equal to 1. Also, the chance to dying is the complementary probability of the chance to survive.

Therefore, based on these considerations, the utility-based multicriteria model constructed for the screening decision problem is described by equations (6)–(8).

(6) $$\begin{array}{c} u\left(\text{ih}\right)=\pi \left(\text{Shi}\right)\left[k_{\text{L}}u\left(\text{Shi}\right)+k_{\text{c}}u\left(\text{Chi}\right)\right]+\left(1-\pi \left(\text{Shi}\right)\right)\left[k_{\text{L}}u\left(\text{Dhi}\right)+k_{\text{c}}U\left(\text{Chi}\right)\right], \end{array}$$

(7) $$\begin{array}{c} u\left(\text{hs}\right)=\pi \left(\text{Shs}\right)\left[k_{\text{L}}u\left(\text{Shs}\right)+k_{\text{c}}u\left(\text{Chs}\right)\right]+\left(1-\pi \left(\text{Shs}\right)\right)\left[k_{\text{L}}u\left(\text{Dhs}\right)+k_{\text{c}}U\left(\text{Chs}\right)\right], \end{array}$$

(8) $$\begin{array}{c} u\left(\text{icu}\right)=\pi \left(\text{Sicu}\right)\left[k_{\text{L}}u\left(\text{Sicu}\right)+k_{\text{c}}u\left(\text{Cicu}\right)\right]+\left(1-\pi \left(\text{Sicu}\right)\right)\left[k_{\text{L}}u\left(\text{Dicu}\right)+k_{\text{c}}U\left(\text{Cicu}\right)\right]. \end{array}$$

As to equations (6)–(8), the physicians receive a recommendation about which alternative is the best one for accommodating the COVID-19 patient. This is the one that presents the highest multiattribute utility function.

In the next section, a practical application of this utility-based multicriteria model is presented in order to illustrate how this mathematical model is used to support the decision-making problem about screening. Also, the decision information system, called SIDTriagem, is presented.

## 4. Practical Applicability and Results

To apply the proposed model for aiding the screening of patients with suspected COVID-19, the physician should first input information about the patient's chances of survival in three scenarios: isolation at home, a hospital stay, or in an intensive care unit (ICU). This information should be given based on the patient's symptoms and clinical state. These chances of survival, however, are not precisely established and involve subjective factors that may not be quantified. Therefore, during the development of the system, three physicians were consulted by an analyst in order to find out what would make them feel more comfortable about providing such information. As a result of this consultation process, it was verified that the physicians preferred to give information about chances of survival on a verbal scale, instead of providing numbers. Thus, a 5-point Likert scale was developed for establishing such probabilities: very low, low, medium, high, and very high. Each of these levels is associated to a probability range of 20% width, which was also calibrated with the physicians. The reason of using probability ranges instead of exact values of probabilities is related to the inherent imprecision and subjectivity of this information.  Table 1 illustrates the association of each level of the scale with the probability ranges.

According to those probability ranges, a Monte-Carlo simulation is performed in order to obtain a recommendation of conduct for the physician. At each simulation instance, a random number between the lower and upper limits of Table 1 is generated for each probability of survival, according to a uniform distribution and taking into account the levels of the verbal scale provided by the user. The recommendation given by the model is based on a robustness index that is computed for each alternative. The robustness index of an alternative is related to the percentage of simulation instances in which the expected overall utility of that alternative is greater than the expected utility of the other alternatives, in accordance with Equations (5)–(7). The model therefore recommends that the user follow the alternative with the largest robustness index.

**Figure 2:**
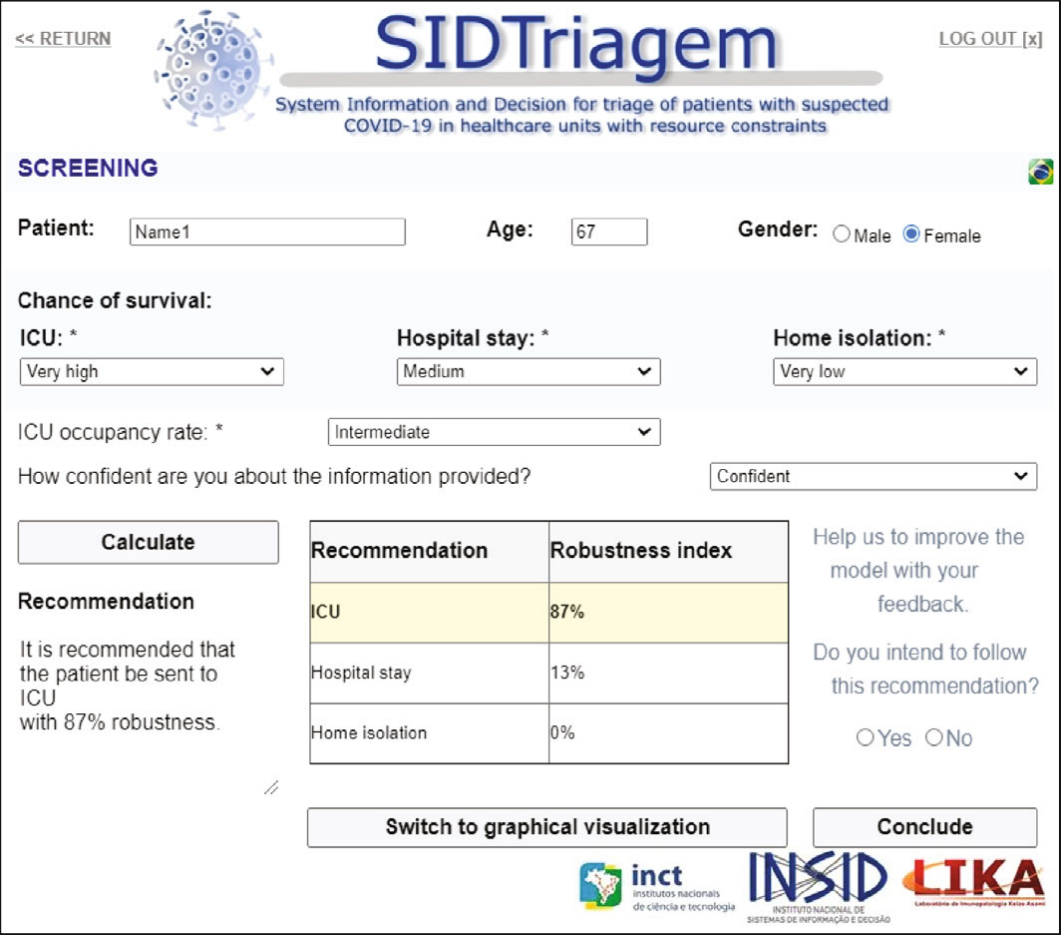
Interface of the SIDTriagem system.

The proposed approach for aiding the screening of patients with suspected COVID-19 is operated by means of a DIS, called SIDTriagem, which is available for users at http://insid.org.br/sidtriagem/app/. Physicians log on to the system and then he/she enters the patient's name, age, and gender (optional data).  Figure 2 shows the interface of the system, with a practical hypothetical example.

In Figure 2, a 67 year-old woman is considered to have been evaluated by the user of the system (a physician) at a healthcare unit. By examining this patient and analyzing all her symptoms and her clinical state, the physician enters information about the chances of survival of this woman in three scenarios: in an ICU, in a hospital ward, and during isolation at home. Let us assume that the physician evaluates her as either *very high* or *medium* or *very low*, respectively. Then, the physician should estimate the ICU occupancy rate at that time, also on a verbal scale: low, intermediate, and high. As previously explained in Section 3.2, this ICU occupancy rate is for calibration of the Fp parameter, which influences the utility of the cost of sending the patient to the ICU. Let us consider that there is intermediate occupancy rate at that moment. Finally, the physician may optimally state how confident he/she is about the information provided: very unconfident, unconfident, neutral, confident, very confident, or even N/A. This information is not used by the mathematical model, but it may be further used in future studies to evaluate the behavior of physicians in such situations.

After entering all the input data, the user clicks on the “Calculate” button and the recommendations obtained based on the simulations are shown to the user. In this case, the recommendation of the system is to send the patient to the ICU, with 87% of robustness. This means that, in 87% of the simulations instances performed, this alternative had the highest expected utility, compared to hospital stay and isolation at home. Hospital stay had a robustness index of 13%, which means that in 13% of the simulation instances this alternative had the greatest expected utility value. The robustness index for isolation at home was 0, indicating that this alternative never wins against the others in terms of expected utility.

**Figure 3:**
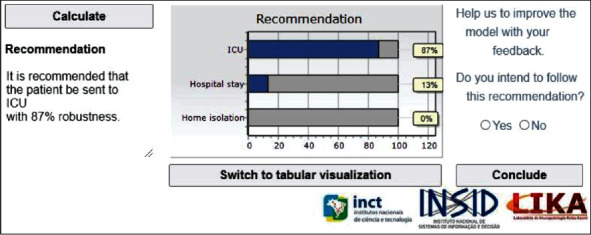
Graphical visualization of the results.

The system also provides user with an alternative way of visualizing the results. By clicking on the “Switch to graphical visualization” button at the bottom of Figure 2, a bar graphic appears as an alternative possible visualization, as Figure 3 shows. These two ways of visualization were included in the system due to the feedback of the physicians; some of whom prefer to visualize them in a table with numbers, and others prefer graphics. Therefore, the system provides both numbers and graphics.

Finally, the user may choose to state whether or not he/she intends to follow the recommendation. Also, there is a space for recording the feedback of the users of the system, by clicking on the “Conclude” button. This feedback helps to make further improvements to the model and to the system itself.

It should be highlighted here that this system should be used as a support tool for aiding screening decisions, based on a structured mathematical model. There are no normative purposes, however, with the use of this system. A recommendation is given, but the final decision always rests with the physician, who should take into account all subjective factors involved in each specific situation.

## 5. Conclusions

In this paper, a utility-based multicriteria model for aiding screening decision situations of patients with suspected COVID-19 was proposed. The screening problem is critical due to the scarcity of treatment resources in hospitals, such as ICU beds, for instance. Therefore, a structured mathematical modeling of this problem is important for aiding physicians to decide if a suspected COVID-19 patient should go to an ICU, a hospital ward, or stay at home in isolation.

The mathematical model was built based on the decision analysis concepts and multiattribute utility theory (MAUT), considering the inherent stochastic nature of this decision-making problem. Considering the inherent imprecision associated to estimating the patient's chances of survival, the proposed model works with probability ranges that serve as an input for a Monte-Carlo simulation model. Moreover, considering the difficulty that physicians have in providing this information due to the subjectivity of the factors involved, a verbal scale is used for estimating patients' chances of survival.

The proposed approach is operated by means of a decision information system, which has a user-friendly interface and can be easily used by physicians in healthcare units worldwide. The information obtained from the occurrences registered in the system is stored in a database. Finally, as suggestions for future research, the occurrences registered in the system would be extremely useful for conducting several kinds of analyses, including a comparative analysis of what the model proposes and what doctors actually do, in practice. Also, behavioral studies based on the data obtained from the physicians' records may be useful for improving the design of decision information systems and their functionalities.

## Figures and Tables

**Table 1:** Ranges of probabilities of survival.

Verbal scale	Lower limit	Upper limit
Very low	0	20%
Low	20%	40%
Medium	40%	60%
High	60%	80%
Very high	80%	100%

## Data Availability

The database generated from the occurrences registered in the SIDTriagem used to support the findings of this study are restricted by the Ethical Committee in Research of the Federal University of Pernambuco with CAAE (“Certificado de Apresentação e Aprecição Ética” - Certificate of Presentation and Ethical Appreciation) number 31065820.5.0000.5208 in order to protect patient privacy. Data are available from the corresponding author upon request according with the criteria for access to confidential data.
